# Visual Measurement of Water Level under Complex Illumination Conditions

**DOI:** 10.3390/s19194141

**Published:** 2019-09-24

**Authors:** Zhen Zhang, Yang Zhou, Haiyun Liu, Lili Zhang, Huibin Wang

**Affiliations:** College of Computer and Information Engineering, Hohai University, Nanjing 211100, China; yangzhou_cies@hhu.edu.cn (Y.Z.); haiyun_liu@hhu.edu.cn (H.L.); lilzhang@hhu.edu.cn (L.Z.); hbwang@hhu.edu.cn (H.W.)

**Keywords:** water-level measurement, machine vision, image processing, flow-measurement system

## Abstract

Image-based water level measurement is a visual-sensing technique which automatically inspects the reading of the water line via image processing instead of the human eye. It can be realized easily on an existing video surveillance system and has advantages like low cost, non-contact, as well as results that are verifiable. It has the potential to be widely used in flood and waterlogging monitoring, while facing the challenge that water-line detection under complex natural or artificial illumination conditions is quite difficult in field applications. To handle this problem, a method is proposed assuming that the water line is generally located on the row with the largest local change of gray or edge features in the image of the water gauge. The water line is determined by coarse-to-fine detection of the position of the maximum mean difference (MMD) of the horizontal projections of gray and edge images. Image-based flow-level measurement systems were developed at two measurement sites. In situ comparative experiments were conducted with the float-type stage gauge and other image-based methods. The results show that the fusion of gray and edge features can overcome the shortcomings of single feature methods under complex illumination conditions such as dim light, glares, shadows and artificial night lighting. A coarse-to-fine strategy utilizes the periodicity of the surface pattern distribution of the standard bicolor water gauge, which improves the reliability of water-line detection. The resolution and accuracy of water-level measurement are 1 mm and 1 cm, respectively. In particular, the MMD value is efficient at identifying extremely unfavorable conditions and reducing gross errors.

## 1. Introduction

Accurate acquisition of hydrological data of natural rivers during a high flood season is important for flood and urban water logging alerts. The water level is one of the most basic hydrological elements in rivers, lakes and reservoirs. The information of water supply, rainstorm, flood discharge, runoff, sediment, nutrient transport rate in cities and irrigation areas usually needs to be calculated according to the measured water level [1,2,3]. All-weather, real-time and automatic water-level monitoring is essential [4]. According to the principle of measurement, existing water-level gauges can be roughly divided into the float-type, the pressure-type, the ultrasonic-type and the radar-type [5,6,7]. However, float-type gauges have large cumulative measurement errors and need to be re-calibrated frequently. The measurement precision of pressure-type gauges is affected by the density of water. The precision of ultrasonic-type gauges is affected by the environment in terms of the temperature, humidity, etc. The radar-type gauges are expensive, and the measurement process is affected by raindrops and snowflakes. There are some limitations in the practical use, installation and daily maintenance of contact equipment, which are easily restricted by external factors such as water quality, temperature and sediment content [8,9]. Therefore, exploring non-contact water level measurement technology is the current research hotspot.

In recent years, a network video surveillance system has gradually become the standard configuration at all levels of hydrological bureaus and stations [10,11]. Ensuring daily monitoring, remote dispatching and disaster alert are important measures to make good use of and protect water resources and water conservancy facilities, which provide favorable conditions for water-level measurement based on video images [12,13,14]. There are also some automatic water-level monitoring systems based on image [15,16,17,18]. Image-based methods use image processing instead of human eyes to detect water line readings automatically, and are divided into the following two kinds:

In methods inspired by human vision, the position of water line is located by detecting graduation lines and recognizing characters [19,20,21,22,23]. Chen et al. [24] proposed character segmentation according to the features of a water gauge surface pattern, using a template matching method to realize character recognition and calculate the water-level reading. But under the condition of insufficient illumination or low image resolution, this cannot be applied in practice. Zhong [25] proposed water-level measurement based on the water graduation line. The graduation line is identified by gray transformation, edge detection, image thinning and K-means clustering analysis. However, the algorithm is sensitive to interference such as water gauge surface contamination, occlusion and damage. Chen [26] used the Hough transform to identify the graduation line, but the standard bicolor water gauge also has strong horizontal characteristics which can easily lead to false detection. The principle of this kind of method is intuitive, but requires high image quality. It depends on the visibility of graduation lines and characters, and is affected by factors such as local fouling and damage to the water gauge, low image resolution, insufficient natural light and the overexposure of artificial light. Under these conditions, there will be gross errors in measurement.

Methods based on machine vision first detect the position of water line in the image and then convert the coordinate transformation relationship into a water-level reading to realize water-level measurement [27,28,29,30]. The horizontal projection method is the most widely used method for standard bicolor water gauge. According to different features, it includes horizontal projection of a gray image [31,32], horizontal projection of a binary image [33] and horizontal projection of an edge image [34]. The position of the water line is determined by searching the abrupt points in the horizontal projection curve. However, under complex illuminations and flow conditions, due to the uneven distribution of gray value affected by random noise such as glares and shadows on the water surface, the abrupt points in the horizontal projection curve may be affected by noise, resulting in an error of the water line position, which makes it difficult to ensure the accuracy of the measurement.

Compared with the method inspired by human vision, this kind of method has the advantage of no detection of graduation lines and characters. Assuming that the gray value of the water gauge is higher than the water surface, the Otsu method [35] is suitable for the case of a large difference in gray distribution. When the gray distribution of the water gauge or water surface is uneven, the global threshold obtained by the Otsu method is not the local optimal segmentation threshold in the water line region, which results in large errors of water line segmentation. The order-statistic filtering (OSF) method [36] sets several sampling points in the water gauge and water-surface image, ascends through sorting with a bubble sort algorithm, then calculates the adaptive segmentation threshold. However, these methods based on single threshold may cause the gray value of a water gauge to be lower than the water surface under complex illumination conditions, which causes the measurement to be sensitive to the threshold.

In view of this, this paper adopts the method based on machine vision, and mainly focused on the detection of the water line under complex illumination conditions. This paper abandons the idea of single threshold image segmentation. Assuming that the water line generally locates on the row with largest local change of gray or edge features in the image of the water gauge, the water line is determined by coarse-to-fine detection of the position of the maximum mean difference (MMD) of the horizontal projections of gray and edge images. Image-based flow level measurement systems were developed and deployed at two measurement sites. In situ comparative experiments were conducted with the float-type stage gauge and other image-based methods. The results show that our system can realize real-time automatic measurement of the water level. Compared with traditional methods, this technique can avoid the constraints of external factors. Compared with other image-based methods, it can solve the measurement problems under complex illumination conditions such as dim light, glares, shadows and artificial night lighting. Section 2 introduces the measurement site and system. Section 3 introduces the MMD method. Section 4 analyses the in-situ experimental results. Section 5 concludes the paper.

## 2. Measurement Site and System

To evaluate the performance of the method and verify that it can be transposed to other measuring situations, image-based flow level measurement systems were developed and deployed at two measurement sites with different orientations of the target-camera vis-à-vis the sun.

### 2.1. Measurement Site 1

**Figure 1:**
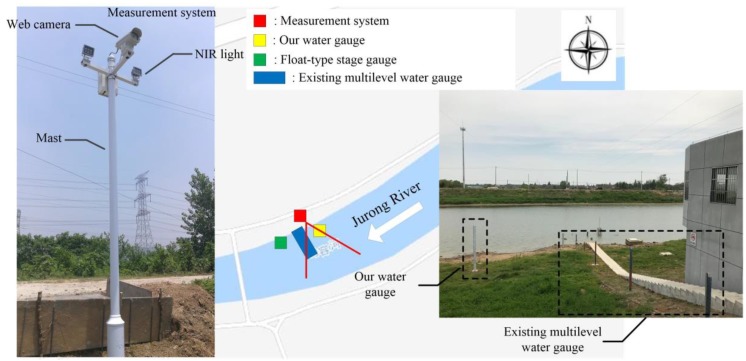
Measurement system at site 1.

Measurement site 1 is located at Qianhancun hydrological station of Jurong River in Nanjing, China, as shown in Figure 1. The station is a national basic hydrological station for monitoring the water level and flood process. The Qinhuai River Basin belongs to hilly and mountainous areas, and runoff is mainly formed by rainfall in the region, mainly in the flood season (May–September). Its runoff accounts for 60–70% of the whole year. Its upper and middle reaches have a small storage capacity, and the flood rises rapidly. Its lower reaches converge into the Yangtze River, and the flood level is affected by the flood and tidal level of the lower reaches of the Yangtze River. There are two inline structures built downstream, and the water level of the station is greatly influenced by its manual operation and control. The measurement reach is straight and the slope is low. The section is U-shaped with a maximum width of 135 m. The station is equipped with a float-type stage gauge (Model NSY WFH–2 Mechanical Encoding Water Level Meter) and 5 water gauges. The stationary datum is used as zero elevation in water-level measurement. The measured maximum water level is 11.60 m (1991) and the measured maximum discharge is 1160 m^3^/s (2003).

The measuring system is mounted on the north bank of river, as shown in Figure 1. Considering the existing multilevel water gauges are made of stainless steel, the contrast in image between water gauge surface pattern and water surface is low, which is not conducive to the detection of the water line. Therefore, our water gauge was deployed at about 5 m upstream. A concrete foundation pile is used to fix a column, on which four monochrome bicolor water gauges of 200 × 1000 mm are installed vertically. The total length is 4 m, and the reading accuracy is 1 cm. The zero elevation of the water gauge is 7.752 m measured by a total station. In practice, it is corrected to 7.968 m by comparing measurement with the float-type stage gauge located at about 10 m downstream. The maximum range is 11.968 m, which covers the highest water level on record. A 4-mega-pixel web camera is integrated inside, facing south to the water gauge with a tilt angle of 12.9°. An optical filter with the pass-band of 850–1050 nm is surface-mounted on the lens to realize near-infrared-imaging. The height from the zero point of the water gauge is 8.4 m and the straight-line distance is 24 m. A 4G router supporting virtual private network (VPN) cloud networking is adopted to realize remote access of the client to the system.

**Figure 2:**
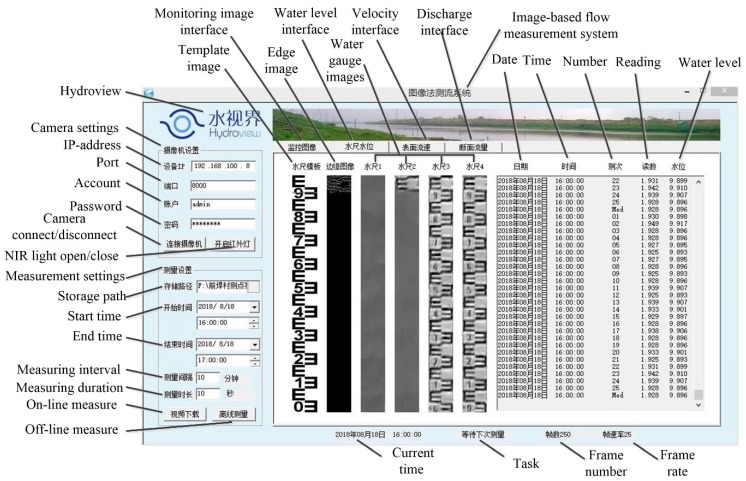
Graphical user interface (GUI) of image-based flow-level measurement software.

The software of the system named Hydroview is developed based on the software development kit (SDK) of a web camera and the Open Source Computer Vision Library (OpenCV). It is used in the above hardware system. The graphical user interface (GUI) of the software is shown in Figure 2. It can automatically execute image processing algorithm to complete the water level measurement, according to the starting and ending time, measurement interval and measurement duration, etc. Two measuring modes are designed: (1) The on-line mode works in a “quasi real-time” way that downloads the local-storaged video files according to a specified time interval from the Micro-SD memory card, rather than captures the real-time video stream. By this means, the influences of network delay and frame loss are improved. (2) The off-line mode is designed for analysis of downloaded historical videos, which is a unique advantage of the image-based method. The measurement output includes not only the measuring results displayed in the text box, but also the water gauge images for visual verification.

### 2.2. Measurement Site 2

The measurement site 2 is a non-gauging site. It locates at the Yanglou Stream of Qinyuan County in Zhejiang Province of China, which is a mountain stream with a gravel bed, rockfill slope and 40 m wide cross-section. There is a small hydropower station 500 m upstream, which releases water from 8:30 to 20:30 every day for electricity generation. The water depth varies from 0.1 m to 0.5 m and most river beds are bare during the dry season, while the flow can rise up to 3 m and be full of floating debris during the rainy season. On account of this, an image-based flow level measurement system is developed to provide on-line video monitoring and real-time hydrologic data for the Lanxi Bridge Reservoir 5 km downstream.

**Figure 3:**
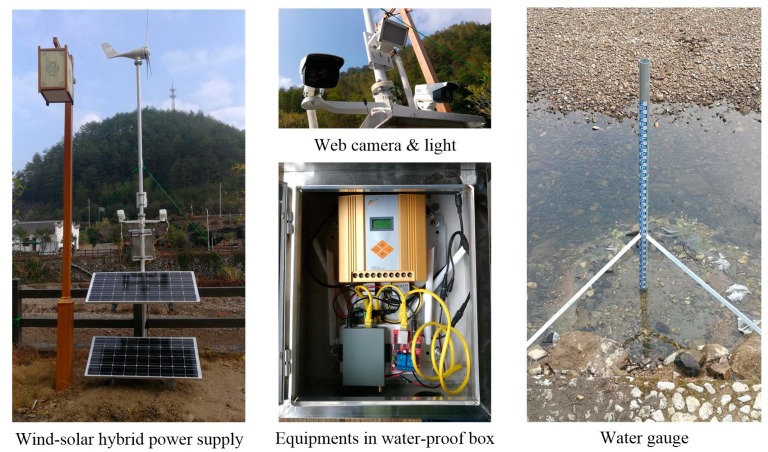
Measurement system at site 2.

The measuring system is mounted on the south bank of river, as shown in Figure 3. Three pieces of standard bicolor water gauge made of an aluminum reflector with the size of 1000 × 80 mm are vertically installed on a stainless steel pillar fixed on river bed. The zero elevation of the water gauge is set to 0 m. A near-infrared imaging web camera with 2 mega-pixel complementary metal oxide semiconductor (CMOS) sensor and 8 mm prime lens is used. As the supplement to the built-in light of camera, a near-infrared light with a light-emitting diode (LED) array at 850 nm wavelength is used for night illumination. A set of wind-solar hybrid power supply is adopted to keep the measurement system continuously running for at least 7 days. See Reference [36] for details.

## 3. Water-Level Measurement

### 3.1. Overview

**Figure 4:**
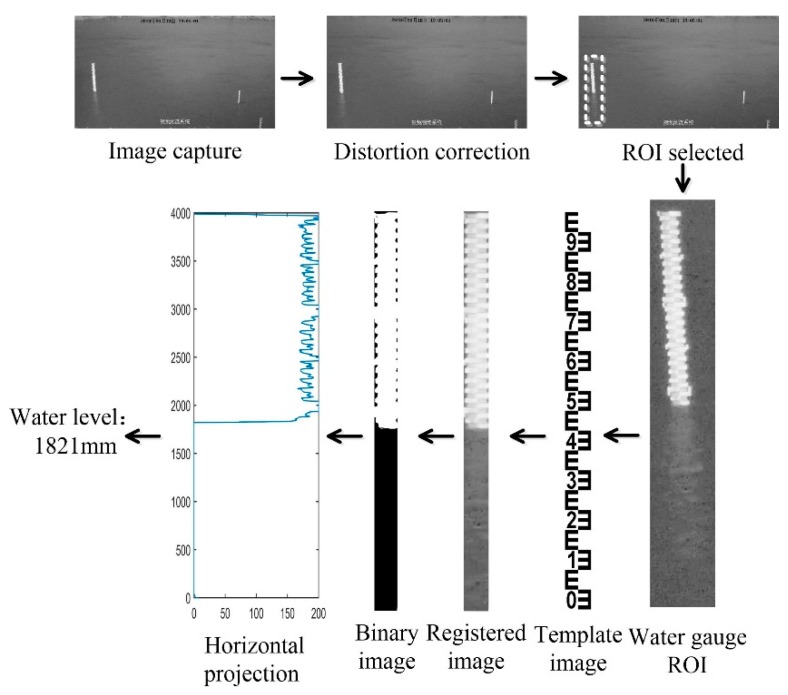
Principle of water-level measurement. (ROI, region of interest).

According to the principle of monocular vision measurement, if the water gauge surface is approximated to a plane, the physical plane and its image on the sensor plane and orthophoto without perspective distortion satisfy the mapping homography relationship [37]. Consequently, the basic idea of water-level measurement based on monocular camera is shown in Figure 4. Firstly, the video image of river surface is captured by monocular camera, and the coordinates of corners are selected to calculate the coordinate transformation matrix in the water-level measurement area. Then, the coordinate transformation matrix is used to correct the image distortion, and the perspective deformed image is registered in the template image to achieve pixel alignment. Next the orthophoto correction image is binarized, and the gray value of each row of pixels is accumulated to obtain the horizontal projection curve. By searching the abrupt change value in the horizontal projection curve, the water line between water gauge and water surface is determined, and then the water level is obtained [36]. This idea of water-level measurement is a two-step process: one detecting the water level on a pixel space, and two translate these in real world coordinates. The MMD method focuses on the detection of the water level on a pixel space, which is the first step.

### 3.2. Basis of Water-Line Detection

**Figure 5:**
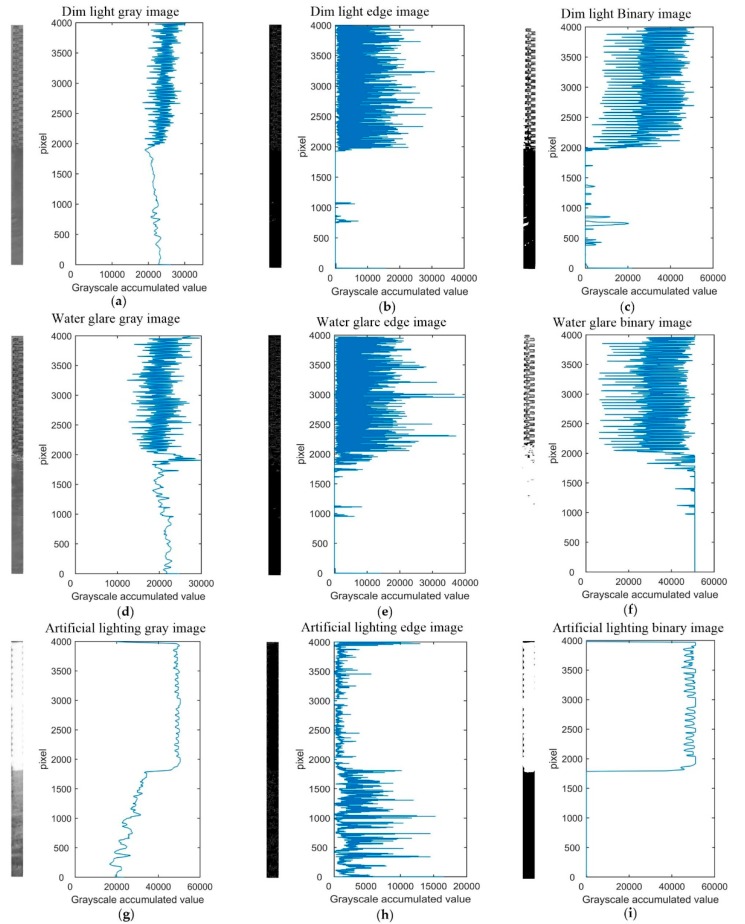
Horizontal projection curves of water gauge orthophotos under three illumination conditions: (**a**) dim light gray image; (**b**) dim light edge image; (**c**) dim light binary image; (**d**) water glare gray image; (**e**) water glare edge image; (**f**) water glare binary image; (**g**) artificial lighting gray image; (**h**) artificial lighting edge image; (**i**) artificial lighting binary image.

The horizontal projection can be divided into three kinds according to different features: gray image, edge image and binary image horizontal projection. The horizontal projection curves of water gauge orthophotos under three illumination conditions are given below. The edge images are generated by a Canny operator and the binary images are generated by the Otsu method, as shown in Figure 5. The position of the water line is determined by searching for the abrupt change points corresponding to the water line in the horizontal projection curve. The results are shown in Table 1.

Under dim light conditions, there is little difference in gray value between water gauge and water surface. The change of gray value at water line is not obvious. In horizontal projection of the edge image, the periodicity of pattern distribution on the surface of the water gauge causes fluctuation of the value, and the non-zero value of the water surface only appears at the wave position. In the binary image, the E-type patterns and numbers on the surface of the water gauge are clearly visible, the binary results of the wave on the water surface are white, and the curve is similar to the edge image.

Under water glare condition, the horizontal projection of the gray image has the largest local change at the water line. The horizontal projection of the edge image is similar to that of dim light. The adaptive threshold of Otsu method is higher in the binary image due to the appearance of water surface flare, which results in the water surface binary value being white.

Under artificial lighting conditions, the horizontal projection value of the gray image is stable at 50,000 in the water gauge area, and decreases to 30,000 at the water line, which has the largest local change. The change of horizontal projection in the edge image is concentrated in the water surface area because the periodicity of the surface pattern distribution of the water gauge is invisible due to artificial night lighting. In a horizontal projection of the binary image, the value of the water gauge area is stable at around 50,000, and the value of the water surface area is all 0. The maximum local variation is at the water line.

### 3.3. Maximum Mean Difference (MMD) Method

**Figure 6:**
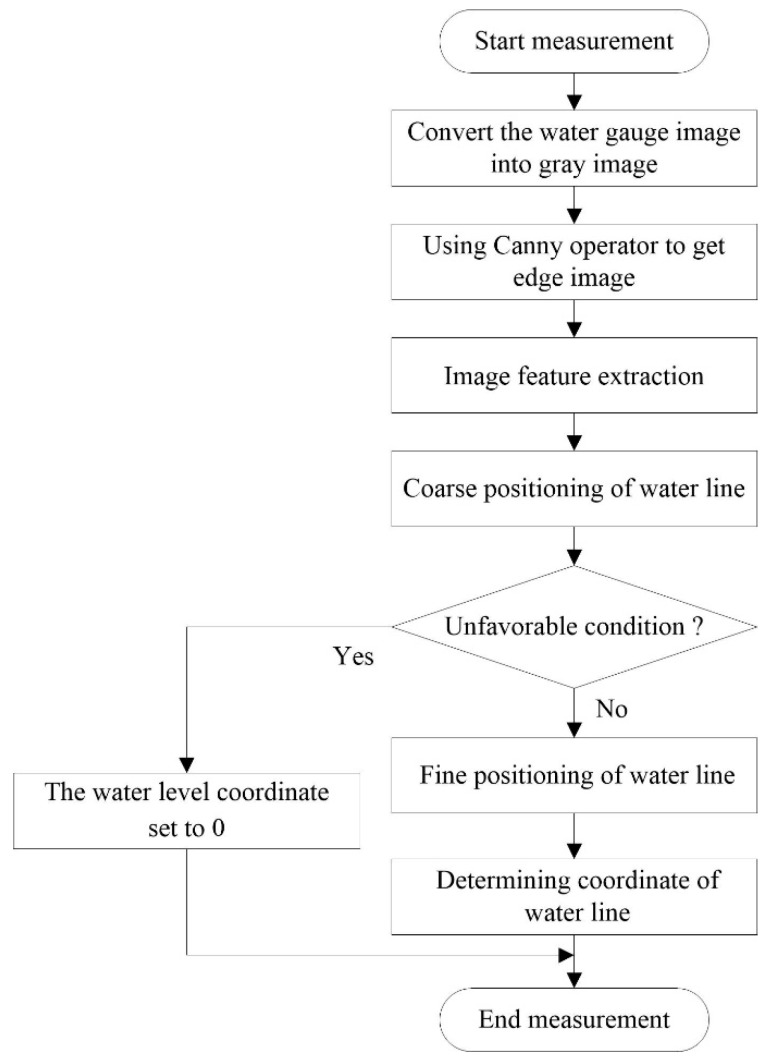
Maximum mean difference (MMD) method flow chart.

The MMD method assumes that the water line generally located on the row with largest local change of gray or edge features in the image of the water gauge. Firstly, the image features are determined by calculating the gray mean horizontal projection of the gray and edge images of the water gauge orthophoto. Then, according to the periodicity of the surface pattern distribution of the standard bicolor water gauge, the position of the maximum mean difference in the horizontal projection curve is searched for as the water line by using the strategy of coarse-to-fine. Specific processes include: set region of interest (ROI) with the period of water gauge image as length. Coarse positioning takes the period of the water gauge image as stepping. The larger observation window can contain more neighborhood information. The heterogeneous area can be identified by searching for the position with maximum value of gray mean difference, which is the candidate region of the water line. After coarse positioning, unfavorable conditions are identified, and fine positioning is carried out without unfavorable conditions. Fine positioning takes 1 pixel as stepping, searching for the position with the maximum value of gray mean difference in the candidate region of the water line. Finally, the physical resolution of the template image can be directly used to convert the water line coordinate to the actual water level. The random errors of multiple measured values are removed by median filtering. The method flow chart is shown in Figure 6.

#### 3.3.1. Image Feature Extraction

The gray image is obtained by gray-scale processing of the water gauge orthophoto with H=4000 pixel length and W=200 pixel width, and then the gray image is transformed into the edge image by the Canny operator. According to Equations (1) and (2), the gray mean horizontal projection of the gray image $Mean_{G}(r)$ and edge image $Mean_{E}(r)$ are calculated respectively:

(1) $$Mean_{G}(r)=\frac{B_{G}\left(r,1\right)+B_{G}\left(r,2\right)+B_{G}\left(r,3\right)+\cdots +B_{G}\left(r,W\right)}{W},$$

(2) $$Mean_{E}(r)=\frac{B_{E}\left(r,1\right)+B_{E}\left(r,2\right)+B_{E}\left(r,3\right)+\cdots +B_{E}\left(r,W\right)}{W},$$

where $B_{G}(r,w)$ is the gray value of the gray image pixel (r,w), $B_{E}(r,w)$ is the gray value of the edge image pixel (r,w), r is the row coordinate of the pixel, w is the column coordinate of the pixel, r=1,2,3,⋯,H, w=1,2,3,⋯,W.

#### 3.3.2. Coarse Positioning of Water Line

**Figure 7:**
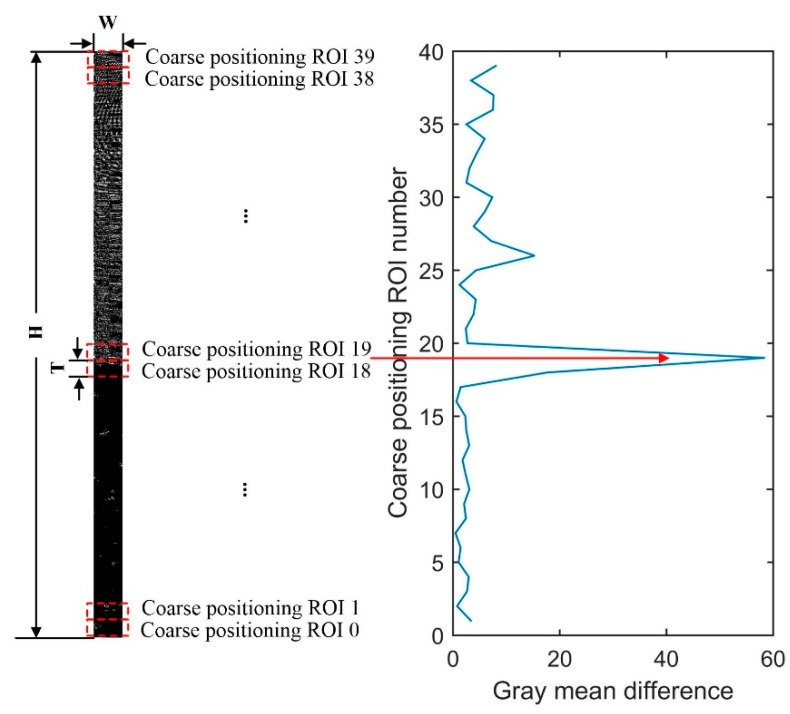
Schematic diagram of coarse positioning of water line.

Coarse positioning ROI is set in the distortion correction image. As shown in Figure 7, the length is T pixel, the width is W pixel, T=100 is the period of standard water gauge image. The top-down ROI number is N,N−1,N−2,⋯,0, N=(H/T)−1=39. According to Equations (3) and (4), gray mean difference of gray image $Diff_{G}(k)$ and edge image $Diff_{E}(k)$ for two adjacent coarse positioning ROI are calculated respectively:

(3) $$Diff_{G}(k)=\left|\frac{\sum_{r=k}^{k+T-1}Mean_{G}(r)}{T}-\frac{\sum_{r=k+T}^{k+2T-1}Mean_{G}(r)}{T}\right|,$$

(4) $$Diff_{E}(k)=\left|\frac{\sum_{r=k}^{k+T-1}Mean_{E}(r)}{T}-\frac{\sum_{r=k+T}^{k+2T-1}Mean_{E}(r)}{T}\right|,$$

where k=1,T,2T,⋯,H−2T represents the coordinates of the pixels, $Mean_{G}(r)$ is the gray mean of line r of the gray image and $Mean_{E}(r)$ is the gray mean of line r of the edge image.

The maximum value of the gray mean difference of the gray image $Diff_{G}(k)$ and edge image $Diff_{E}(k)$ for two adjacent coarse positioning ROI is taken as the gray mean difference Diff(k):

(5) $$Diff(k)=\max\left(Diff_{G}(k),Diff_{E}(k)\right).$$

The maximum value of the gray mean difference Diff(k) is $Diff(k^{\prime })$, and $k^{\prime }$ is the row coordinate of the pixels corresponding to the maximum value Diff(k).

#### 3.3.3. Identification of Unfavorable Condition

**Figure 8:**
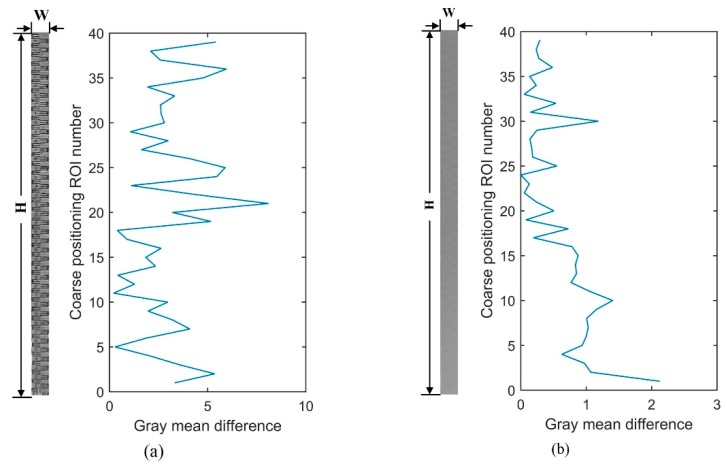
Gray mean difference of coarse positioning in unfavorable conditions: (**a**) out range; (**b**) poor visibility.

Set S=10 as the detection threshold for an unfavorable condition. This detection threshold S is an empirical threshold based on in situ measurement data under different illumination conditions at multiple measuring sites. $Diff(k^{\prime })<S$ indicates that the detection condition is not satisfied, the water level coordinate l is set to 0 and the detection is completed. The gray mean difference of coarse positioning is calculated under the condition of out range and poor visibility. The result is shown in Figure 8. The maximum difference of gray mean value of coarse positioning is 8.096, which is less than the detection threshold. It is judged to be in an unfavorable condition and the water level coordinate is assigned to 0. In the case of poor visibility, due to extreme weather, it is difficult to distinguish the water gauge and water surface. Manual reading is also very difficult in this case. The maximum difference of gray mean value of coarse positioning is 2.117, which is less than the detection threshold, too. It is also judged to be in an unfavorable condition.

#### 3.3.4. Fine Positioning of Water Line

**Figure 9:**
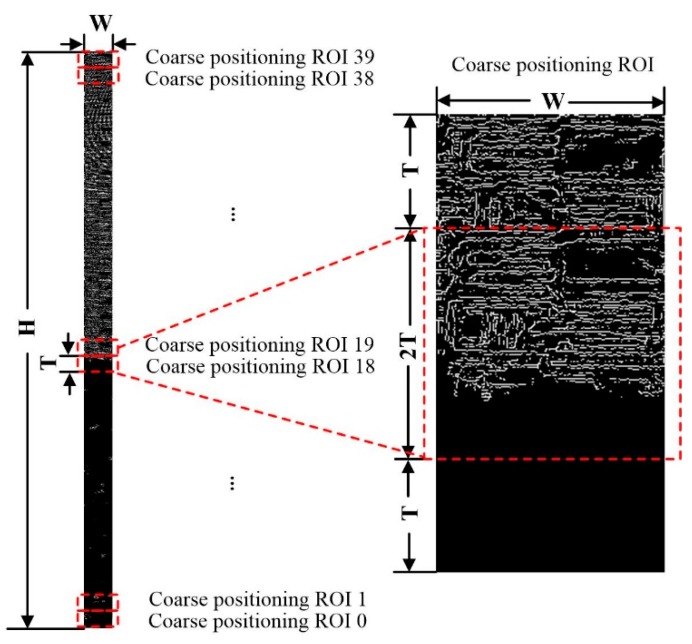
Coarse positioning ROI of water line.

**Figure 10:**
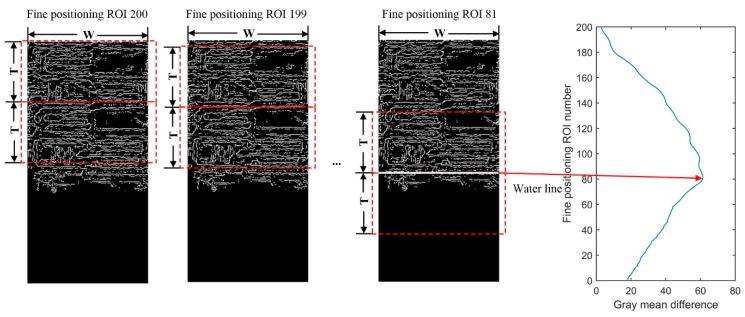
Schematic diagram of fine positioning of the water line.

Two adjacent coarse positioning ROIs with the largest gray mean difference Diff(k) are selected as the coarse positioning ROI of the water line. The length is 2T pixel, the width is W pixel, and the top row coordinate is $k^{\prime }$, as shown in Figure 9. In coarse positioning ROI, feature fusion is used to precisely locate the water line, and set up fine positioning ROI as shown in Figure 10. The length is 2T pixel and the width is W pixel. They are divided into upper and lower half areas of the same size. The length is T pixel and the width is W pixel. They are stepped by a single pixel and numbered n,n−1,n−2,⋯,1 from top to bottom, where n=2T=200. According to Equations (6) and (7), the gray mean difference of the gray image $Diff_{G}(k_{1})$ and edge image $Diff_{E}(k_{1})$ in the upper and lower half of fine positioning ROI is calculated respectively:

(6) $$Diff_{G}(k_{1})=\left|\frac{\sum_{r=k_{1}}^{k_{1}+T-1}Mean_{G}(r)}{T}-\frac{\sum_{r=k_{1}+T}^{k_{1}+2T-1}Mean_{G}(r)}{T}\right|,$$

(7) $$Diff_{E}(k_{1})=\left|\frac{\sum_{r=k_{1}}^{k_{1}+T-1}Mean_{E}(r)}{T}-\frac{\sum_{r=k_{1}+T}^{k_{1}+2T-1}Mean_{E}(r)}{T}\right|,$$

where $k_{1}=k^{\prime }-T+1,k^{\prime }-T+1,k^{\prime }-T+2,\cdots ,k^{\prime }+T$ represents the row coordinates of the pixels.

The maximum value of the gray mean difference of the gray image $Diff_{G}(k_{1})$ and edge image $Diff_{E}(k_{1})$ for fine positioning of the upper and lower half of the ROI is taken as the gray mean difference $Diff(k_{1})$:

(8) $$Diff(k_{1})=\max\left(Diff_{G}(k_{1}),Diff_{E}(k_{1})\right).$$

The maximum value of gray mean difference $Diff(k_{1})$ is $Diff({k_{1}}^{\prime })$, and ${k_{1}}^{\prime }$ is the row coordinate of the pixels corresponding to the maximum value $Diff(k_{1})$.

#### 3.3.5. Determining Coordinate of Water Line

The coordinate of the water line is determined to be $l={k_{1}}^{\prime }+T-1$ pixel. Because the distortion correction image and the template image are in a unified coordinate system, the physical resolution of the template image Δd can be directly used to convert the water line coordinate to the actual water level L=l/Δd, and the measurement process is completed. Here set Δd as 1 pixel/mm, corresponding to the water level measurement resolution of 1 mm.

## 4. Experimental Results

### 4.1. Experiment in Measurement Site 1

**Figure 11:**
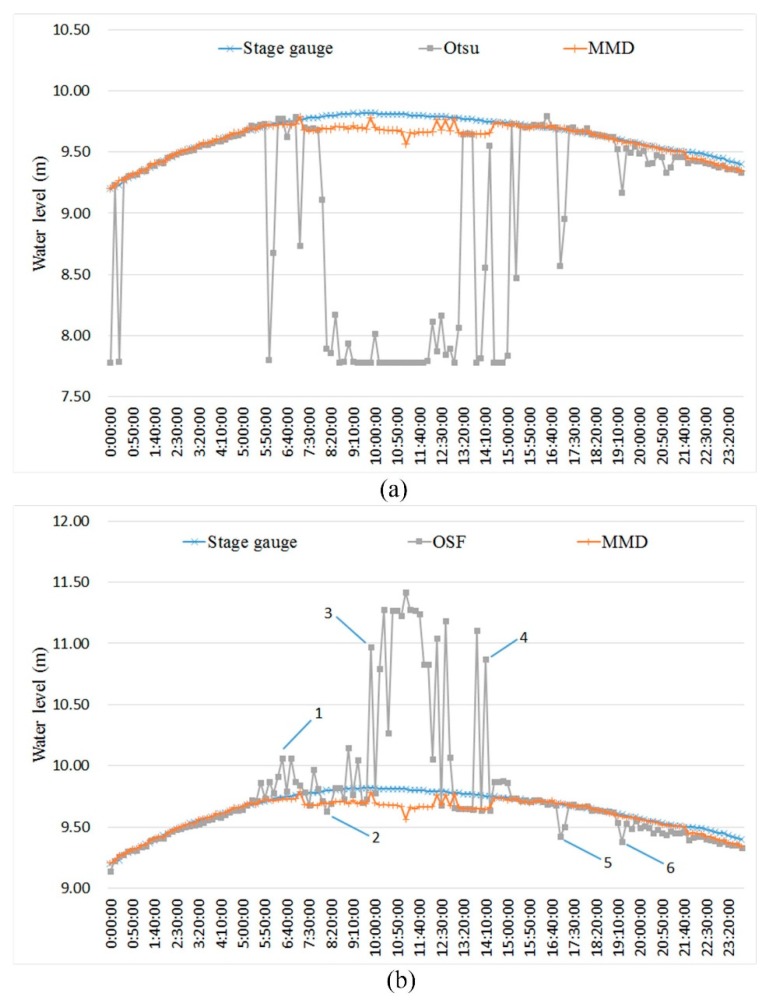
Water-level measurement results: (**a**) comparison results of Otsu and MMD methods; (**b**) comparison results of the order-statistic filtering (OSF) and MMD methods.

Since the system deployed in July 2018, we have carried out the long-term in situ comparative experiments. In view of the main research contents of this paper, the most typical 24-h measurements during the typhoon period are given, which shows that the system can be applied to complex illumination conditions to verify the effectiveness of the method. The experimental date was 18 August 2018, during which Typhoon Rumbia passed through and increased the water level. The weather changed from cloudy to sunny, and the wind was a moderate breeze. The measured data of the float-type stage gauge in the hydrology station are taken as the reference. In order to verify the uncertainty of the MMD method under complex illumination conditions, the water-level measurements of images collected under different illumination conditions were carried out, and the results were compared with two adaptive threshold methods based on Otsu and OSF. The continuous measurement interval was set to 10 min, and 144 data obtained from 0:00:00 to 23:50:00 were selected. The measurement results are shown in Figure 11. Hydrological station float-type stage gauge data recorded the changes of water level in the day, ranging from 9.2 m to 9.8 m. During 7:10:00–15:50:00, the Otsu method resulted in errors when illumination conditions changed, and the measured value was significantly lower than the reference value, as shown in Figure 11a. The results of the OSF method were better than those of the Otsu method, but the measured value was higher than the reference value, as shown in Figure 11b. During 7:10:00–14:40:00 and 21:50:00–23:50:00, the results of the MMD method were slightly lower than the reference values, and the errors were about 0.12 m and 0.05 m, respectively. Looking at the water gauge images, we can see that there are aquatic weeds winding around the water gauge, which results in errors. This also affects the threshold selection of the Otsu method and the OSF method.

After removing the winding period of aquatic weed, 85 groups of valid measurement data were left. The gross errors larger than the 0.1 m number (N_E>0.1_) and gross errors larger than the 0.02 m number (N_E>0.02_) of the Otsu, OSF and MMD methods are shown in Table 2, corresponding effective data ratios of Otsu, OSF and MMD methods are 55.3%, 52.9% and 90.6%, respectively. The root mean square error (RMSE) of the MMD method was only 0.0118 m, which was much lower than other two methods. Due to the camera vibration caused by wind, the error of image registration was ±5 cm, which was reduced to ±2 cm by median filtering. These errors are related to the conversion of the actual water level, which can be corrected by a strategy of detecting camera movement and adjusting the exterior orientation parameters [13].

**Figure 12:**
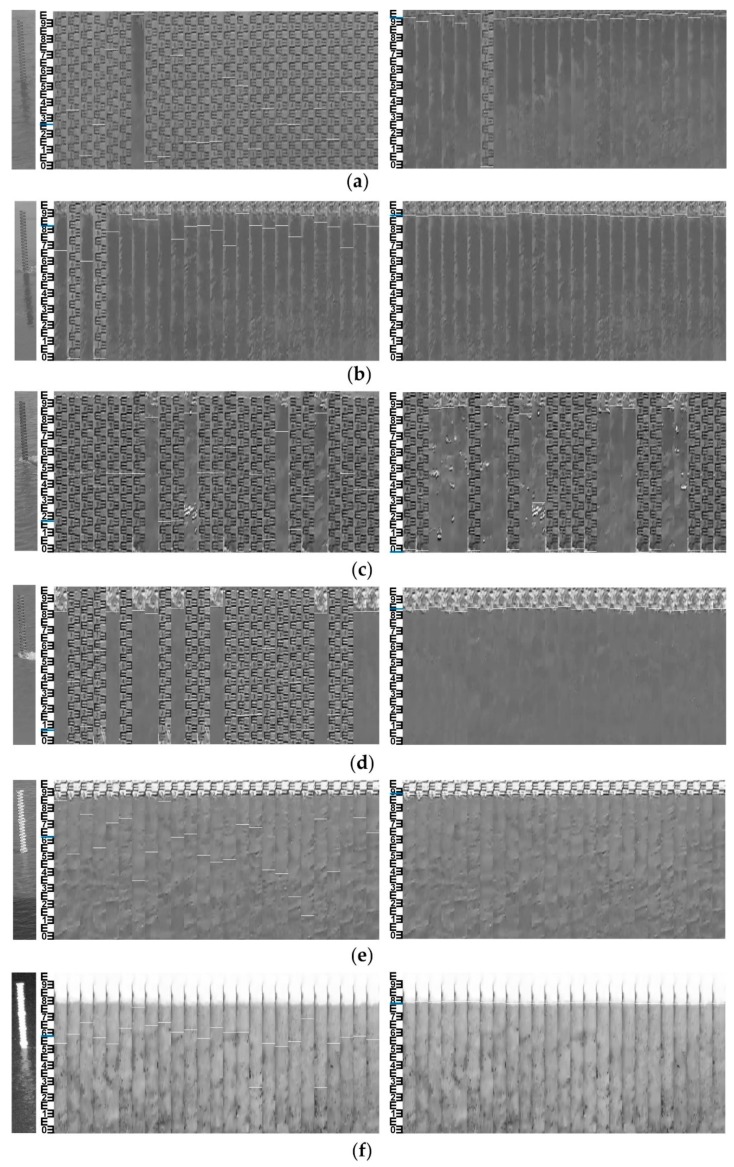
The original water gauge images (left) and visualized OSF (middle) and MMD (right) measurements: (**a**) 6:30:00 dim light; (**b**) 8:10:00 shadow projection; (**c**) 9:50:00 water glare; (**d**) 14:10:00 direct sunlight; (**e**) 17:00 lateral sunlight; (**f**) 19:20:00 artificial lighting.

**Figure 13:**
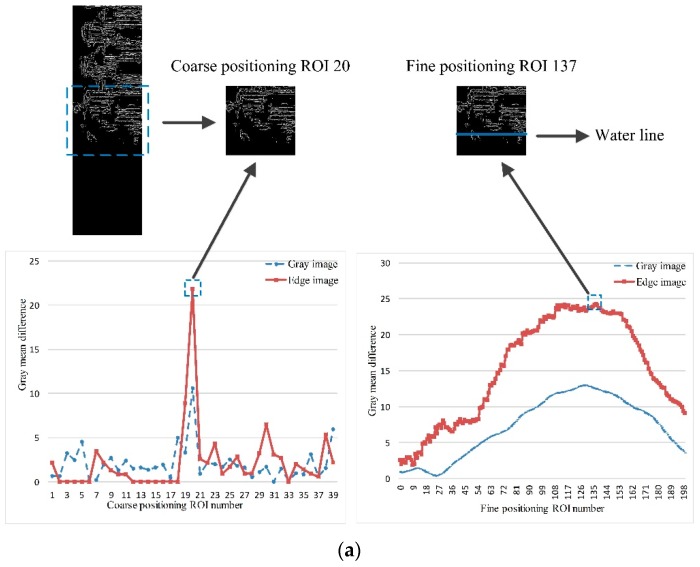

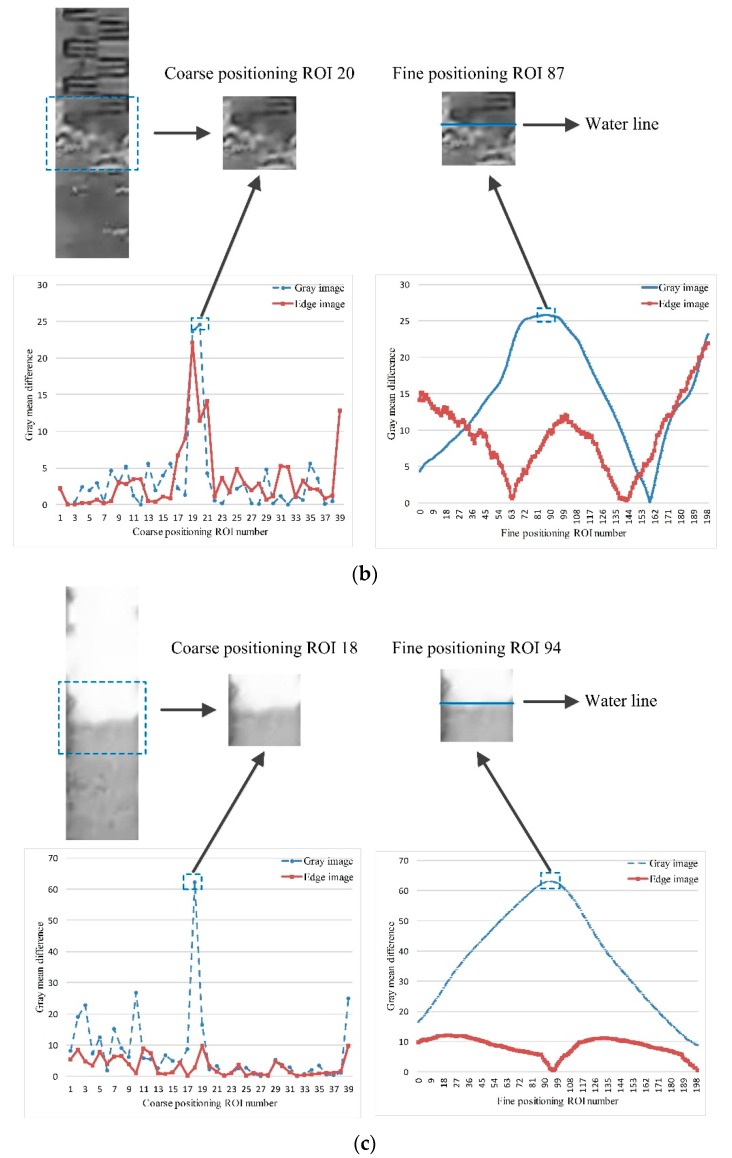
The result of coarse and fine positioning of the water level by the MMD method under three illumination conditions: (**a**) 6:30:00 dim light; (**b**) 9:50:00 water glare; (**c**) 19:20:00 artificial lighting.

In order to analyze the applicability under complex illumination conditions, original water gauge images (left) and visualized water-level measurements of the OSF (middle) and MMD (right) methods under different illumination conditions at 6 moments were selected, as shown in Figure 12. The measuring time is shown in the label 1–6 labeled in Figure 11b. The blue line drawn on the template image represents the median value of the measured water level, corresponding to the white line drawn on the 25 corrected water-level ROI. Table 3 gives the ROI and water-level results of the MMD method under different illumination conditions at 6 moments. Coarse positioning ROI number (gray image) is the ROI number with the maximum gray mean difference of gray image in the coarse positioning process and coarse positioning ROI number (edge image) is the ROI number of edge image. After comparing the former two, the group with the larger values of gray mean difference is selected, and its ROI number is set as coarse positioning ROI number, which can be seen in Figure 13. Fine positioning ROI number (gray image) is the ROI number with the maximum gray mean difference of the gray image in the fine-positioning process and the fine-positioning ROI number (edge image) is the ROI number of the edge image. After comparing the former two, the group with the larger values of gray mean difference is selected, and its ROI number is set as the fine positioning ROI number, which can be seen in Figure 13.

The dim light corresponds to label 1 in Figure 11b. The illumination condition at the junction of morning and dusk is poor, and there is no artificial light supplement. The gray values of the water gauge and water surface are close to each other, which reduces the image contrast and improves the sensitivity of the adaptive threshold. As a result, the water line detected by the OSF method appears on the water gauge after binarization of the water gauge image. The MMD method calculates the gray image mean difference and edge image mean difference of adjacent coarse and fine positioning ROI. When gray image features cannot accurately distinguish the water gauge and water body, the extreme points of mean difference can be accurately detected by using edge image features. As shown in Figure 13a, the water gauge and water are distinguished effectively. The absolute error is 0.011 m compared with the result of the stage gauge. This avoids the wrong measurement of water level when gray values are similar.

The condition of water glare corresponds to label 3 in Figure 11b. The uneven distribution of gray level caused by the local water glare leads to the higher threshold selected by the OSF method, which results in the water surface binary value being white. The MMD method detects extreme points in the gray mean difference of the gray image. As shown in Figure 13b, in fine positioning the maximum of gray mean difference of the gray image is ROI 87, while the maximum of the edge image is ROI 199. After taking the maximum of both, the water line is located at ROI 87, which proves that the MMD method can avoid the problem of false detection of the single feature detection method. Because of the winding of aquatic weeds, the error of water level measured by MMD method is 0.038 m. The valley of the gray mean difference of edge image at ROI 64 and 144 is also the texture difference caused by aquatic weeds.

Artificial lighting corresponds to label 6 in Figure 11b. At night, strong artificial light supplement forms relatively stable, uniform and high-contrast illumination conditions, which are conducive to the measurement of water level. As shown in Figure 13c, the gray mean difference of the gray image in coarse and fine positioning appears at maximum value in ROI 18 and ROI 94 respectively, which is much larger than the maximum value of the edge image. The MMD method can locate the water line accurately and the result is stable. The gray mean difference of the edge image appears as the valley value at ROI 96 because there is strong edge information at the water line, but it does not affect the result of fine positioning.

Note that the difference between the OSF method and MMD method is that: (1) the reflective characteristics of water body are different. The water body of OSF application is clear, and the sediment content of water body measured by MMD method is higher. (2) The material of the water gauge is different. The water gauge used in OSF is made of aluminium alloy, which has strong reflectivity in the near-infrared band and invisible characters in daytime. The MMD method water gauge is made of stainless steel, and the characters are visible in the near-infrared band in daytime. (3) The distance between the water gauge and the camera is different: OSF is 3 m and MMD is 24 m.

### 4.2. Experiment in Measurement Site 2

The MMD method was systematically tested at measurement site 2 during 7 days with typical weather conditions as shown in Table 4. The measuring interval was set as 10 min to capture 144 sets of data every day. Data missing after 13:00:00 in 18–03–09 was caused by an accidental shutdown of the remote-monitoring PC. Fortunately, this deficiency was supplemented by data of 18–03–10 with the same weather condition.

**Figure 14:**
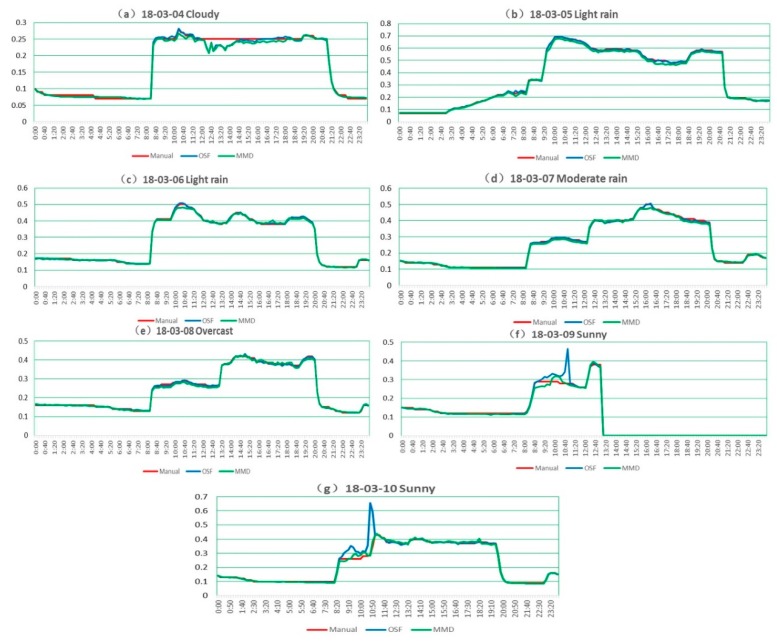
Time series of daily water-level measurements: (**a**) cloudy (18–03–04); (**b**) light rain (18–03–05); (**c**) light rain (18–03–06); (**d**) moderate rain (18–03–07); (**e**) overcast (18–03–08); (**f**) sunny (18–03–09); (**g**) sunny (18–03–10).

As shown in Figure 14, manual observations served as the reference values and the OSF method is taken for comparison. MMD and OSF measurements in Figure 14a show slight fluctuations during 12:20:00–13:40:00. Through the manual inspection of original videos, we find that it is caused by shaking of the camera due to gust influence, making water level bias up to 0.05 m. Figure 14b–e introduce the flow confluence process produced by rain. The influence of light to moderate rain is found to be almost negligible owing to high signal–noise ratio of near-infrared imaging. Measurements in Figure 14f,g show greater uncertainties than others. The reason can be explained that the influence of ambient lighting to optical imaging in sunny days is larger than that in overcast and rainy days.

Table 5 indicates N_E>0.0__2_ of the MMD method and OSF methods, corresponding to effective data ratios of 95.86% and 96.39%, respectively. In particular, the gross errors larger than 0.1 m (observed around 18–03–10 10:40:00) of the OSF method were also induced by shadows of the support frame projected on the water gauge, but the MMD method effectively solved this problem by fusing gray and edge features. In the case of shadow interference, the RMSE of the MMD method was around 0.01 m, while the RMSE of OSF method was around 0.04 m.

**Figure 15:**
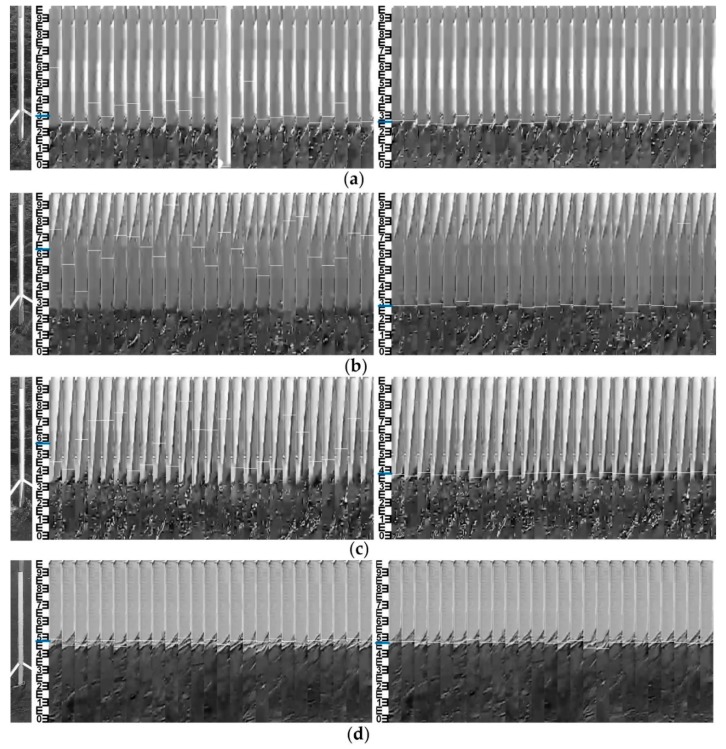
The original water gauge images (left) and visualized OSF (middle) and MMD (right) measurements: (**a**) strong lateral sunlight (09:10:00); (**b**) shadow projection (10:40:00); (**c**) strong direct sunlight (10:50:00); (**d**) high velocity flow (16:40:00).

Compared with site 1, the situation of the camera facing north to the water gauge suffered more from the influence of direct and lateral sunlight, which created more complicated illumination conditions in sunny days. Shadow projection (18–03–10 10:40:00) on the water gauge had similar grayscale with the water surface, making it difficult to distinguish with segmentation methods based on a single threshold. These shadows on the water gauge not only interfered with the calculation of threshold, but also formed residual noises after image segmentation. Strong lateral sunlight (18–03–10 09:10:00) will form horizontal uneven illumination on the water gauge. Glares on water surface and reflections from floating debris randomly generated by strong direct sunlight (18–03–10 10:50:00) were the main adverse factors in the sunny day. For better analyzing the suitability of methods, original water gauge images (left) and visualized water-level measurements of the OSF (middle) and MMD (right) methods under the above three typical illumination conditions are listed and shown in Figure 15. Compared with OSF, which relies on a single gray feature, MMD performs better. The location accuracy of the water line is improved by taking the maximum difference of local image features as the judging condition. 

Note that the reason why the MMD’s RMSE is larger than that of OSF in 3 rainy days is that the water line was inclined due to the high velocity flow against the pillar (18–03–05 16:40:00), as shown in Figure 15d. The manual observation takes the midpoint of the inclined water line as reference value, while the MMD method mainly detects the lower part, which causes gross errors larger than 0.02 m. In this case, the reference value should be determined by comparison with other water-level gauges.

## 5. Conclusions

In order to solve the problem of image-based water level measurement under complex illumination conditions, a water line detection method based on maximum mean difference (MMD) of gray and edge features is proposed. Systematic experiments were conducted at two measurement sites. The results show that: (1) The fusion of gray and edge features can effectively overcome the shortcomings of traditional single-feature detection methods under dim light, water glare, shadow projection and other complex illumination conditions. (2) The coarse-to-fine strategy of searching the maximum mean difference in the horizontal projection is designed to detect the water line according to the periodicity of the surface pattern distribution of the standard bicolor water gauge. The water-level accuracy can reach 1 cm, and effective data ratios up to 90%. (3) The identification of unfavorable conditions is realized. Future work will focus on the identification of floating debris winding around the water gauge during the high flood period and reducing the impact of invariable vibration and slight movement of the camera caused by wind.

## Figures and Tables

**Table 1:** Detection results of water line position in water gauge orthophotos under three illumination conditions (unit: pixel).

Illumination Conditions	Dim Light	Water Glare	Artificial Lighting
Gray Image	2684	1898	1801
Edge Image	3992	3999	3958
Binary Image	1990	0	1788
Manual Reading	1964	1906	1836

**Table 2:** Root mean square errors (RMSEs) and error measurements of Otsu, OSF and MMD methods.

Methods	Otsu	OSF	MMD
RMSE/m	0.4867	0.0818	0.0118
N_E>0.1_	15	12	0
N_E>0.02_	38	40	8

**Table 3:** ROI and water-level results of the MMD method under different illumination conditions.

Illumination Conditions	Dim Light	Shadow Projection	Water Glare	Direct Sunlight	Lateral Sunlight	Artificial Lighting
Coarse Positioning ROI Number (Gray Image)	20	19	20	19	19	18
Coarse Positioning ROI Number (Edge Image)	20	19	19	18	19	39
Coarse Positioning ROI Number	20	19	20	19	19	18
Fine Positioning ROI Number (Gray Image)	129	112	87	133	84	94
Fine Positioning ROI Number (Edge Image)	137	99	199	158	105	21
Fine Positioning ROI Number	137	99	87	133	84	94
MMD Method Water Level Result/m	9.729	9.690	9.782	9.644	9.694	9.588
Stage Gauge Result/m	9.74	9.80	9.82	9.75	9.69	9.60
Absolute Error/m	0.011	0.11	0.038	0.106	0.004	0.012

**Table 4:** Weather conditions during the experiment.

Data	18–03–04	18–03–05	18–03–06	18–03–07	18–03–08	18–03–09	18–03–10
Weather	Cloudy	Light Rain	Light Rain	Moderate Rain	Overcast	Sunny	Sunny
Wind Scale	Scale 1	Scale 5	Scale 3	Scale 2	Scale 1	Scale 1	Scale 2

**Table 5:** RMSEs and numbers of error measurements of the MMD and OSF methods.

Methods	Data	18–03–04	18–03–05	18–03–06	18–03–07	18–03–08	18–03–09	18–03–10
MMD	RMSE/m	0.009	0.012	0.006	0.008	0.006	0.011	0.009
	N_E>0.__1_	0	0	0	0	0	0	0
	N_E>0.0__2_	7	10	3	4	0	8	7
OSF	RMSE/m	0.008	0.004	0.003	0.004	0.004	0.018	0.040
	N_E>0.__1_	0	0	0	0	0	1	2
	N_E>0.0__2_	7	3	0	1	0	10	13
